# Salivary Gland Heterotopia in the Gastroesophageal Junction: A Case Series and Review of the Literature

**DOI:** 10.1155/2018/6078581

**Published:** 2018-09-30

**Authors:** Lina Abdul Karim, Dong Hyang Kwon, Metin Ozdemirli

**Affiliations:** Medstar Georgetown University Hospital, Washington, DC, USA

## Abstract

Heterotopia is defined as the presence of mature, histologically normal, tissue in unusual anatomic sites. When this heterotopic tissue forms a mass, it is called a choristoma. This case series describes 3 cases of gastroesophageal junction (GEJ) salivary heterotopias. While heterotopias are usually incidental findings, choristomas can clinically and endoscopically mimic carcinomas and might lead to unnecessary procedures for the patients. Clinicians should therefore be aware of this entity. Literature search, however, failed to show any reports of salivary gland heterotopias in the GEJ. In fact, literature review revealed only 6 reported cases of salivary gland choristoma in the gastrointestinal tract, none at the GEJ. In this case series, we report 2 cases of salivary gland heterotopia and one case of salivary gland choristoma arising at the GE junction. To our knowledge, this is the first series of its kind in the literature.

## 1. Introduction

Heterotopia is defined as the presence of mature, histologically normal, tissue found in unusual anatomic sites. A mass formed by heterotopic tissue is called a choristoma. Salivary gland choristoma was recognized as early as 1967 by Youngs and Scofield in lower neck [1]. Subsequently more cases of salivary choristoma have been reported mainly in the head and neck region, including the tonsil [2], periparotid and cervical lymph nodes [3], pituitary [4], external auditory canal, thyroglossal duct cyst, mastoid bone, middle ear, tongue, sternoclavicular joint, thyroid and parathyroid glands, and the upper and lower neck regions [5].

Despite common occurrence in the head and neck, reports of salivary gland heterotopia or choristoma in the gastrointestinal tract are scarce. Salivary gland choristoma found in conjunction with gastric heterotopia was first described by Shindo et al. in 1972 [6]. Our literature search revealed only five additional reported cases in the GI since then. The reported anatomic sites include rectal and perianal regions [6–8], descending colon [9], jejunum [10], and esophagus [11]. In this case series, we report 2 cases of salivary gland heterotopia and one case of salivary gland choristoma arising at the GE junction (Table 1). To our knowledge, this is the first series of its kind in the literature.

## 2. Case Report

### 2.1. Case 1

**Figure 1:**
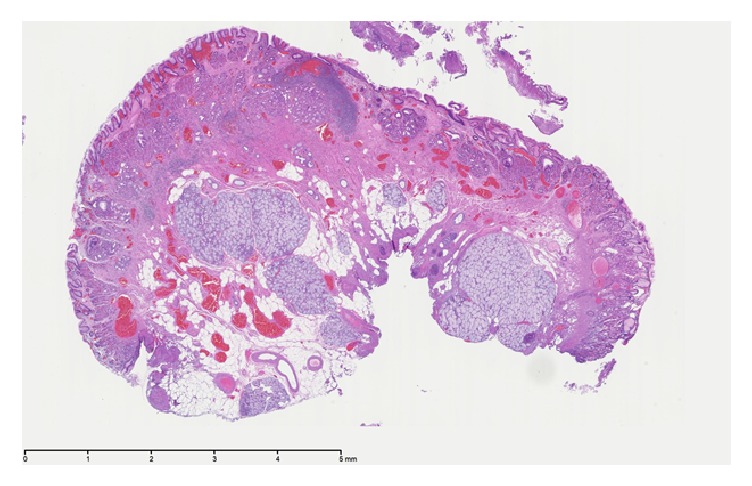
Low-magnification appearance of the heterotopic salivary glands demonstrating their submucosal location surrounded by prominent adipose tissue and congested vessels. This case is classified as a salivary gland choristoma since the heterotopic tissue formed a mass.

**Figure 2:**
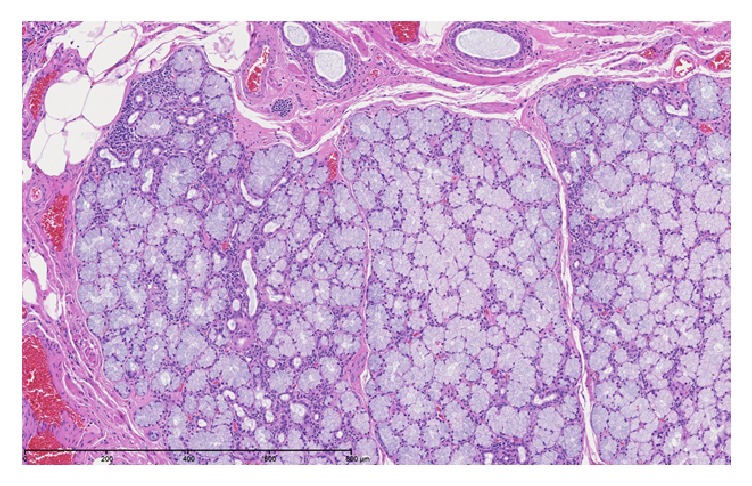
High-magnification appearance of the heterotopic salivary glands demonstrating prominent mucus glands with chronic inflammation.

A 44-year-old Caucasian male presented with an 8-month history of reflux and heart burn, which was relieved by Esomeprazole. Endoscopic evaluation showed a polypoid shaped mass measuring 1 cm in size at the gastroesophageal junction. Ultrasonic evaluation revealed a hypoechoic lesion that was confined to the deep mucosa and submucosa with no deeper layer involvement. The nodule was resected via the endoscopic mucosal resection technique (EMR). Grossly, the specimen was a 1 cm GEJ nodule. It was a single irregular fragment of tan-pink soft tissue that was bisected and entirely processed for microscopy. Microscopic evaluation showed squamous mucosa with oxyntic-type mucosa with moderate chronic inflammation, ectatic vessels, congestion in the mucosa, and a few lymphoid aggregates. In addition, there were prominent mucus glands with chronic inflammation consistent with heterotopic salivary glands, surrounded by prominent adipose tissue and congested vessels in the submucosa (Figures 1 and 2).

### 2.2. Case 2

A 62-year-old Caucasian male with a history of hypothyroidism, Sjogren's syndrome, Raynaud syndrome, chronic GERD, and grade 3 esophagitis presented for an upper endoscopy for the evaluation of suspected Barrett's esophagus. Endoscopic evaluation and barium swallow showed a dilated esophagus. The z-line appeared slightly irregular and there were 2 small islands of salmon colored mucosal lesions immediately proximal to the GE junction.

**Figure 3:**
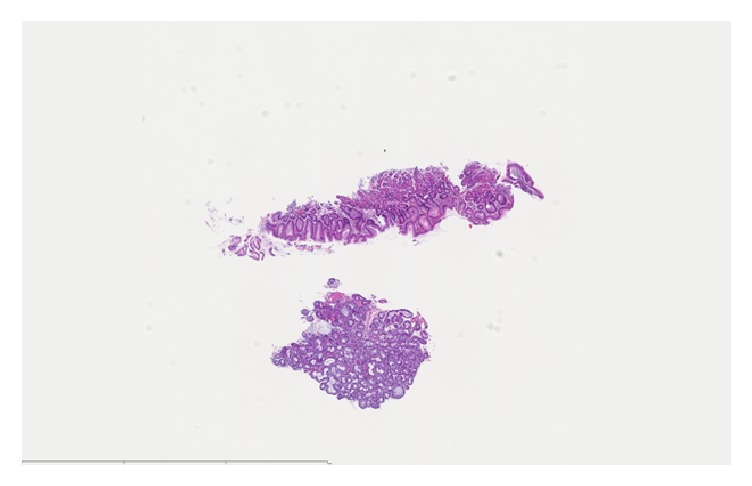
Low-magnification appearance of the heterotopic salivary gland tissue that was received as detached fragments.

**Figure 4:**
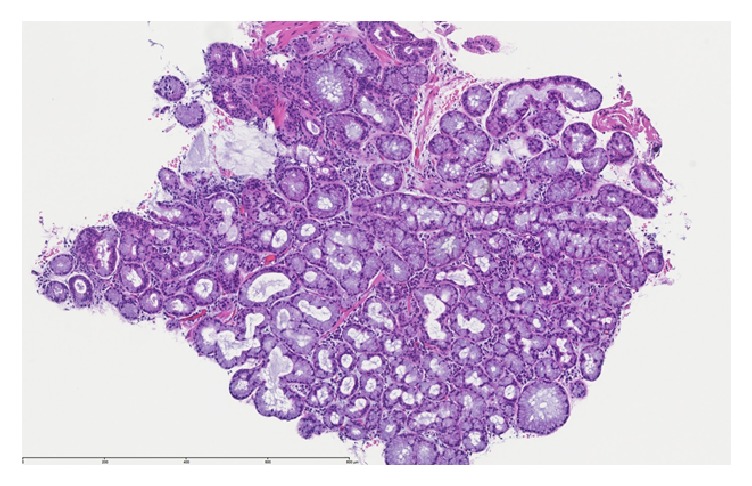
High-magnification appearance of the salivary gland type glandular tissue showing chronic inflammation.

Grossly, the specimen consisted of one piece of tan-white soft tissue, measuring < 1cm. Microscopic evaluation showed fragments of squamous mucosa with focal mild acute erosive esophagitis and basal hyperplasia that was consistent with reflux. In addition, detached fragments of salivary gland type glandular tissue with chronic inflammation, consistent with heterotopic salivary gland tissue, were seen (Figures 3 and 4).

### 2.3. Case 3

A 72-year-old Caucasian male with past medical history of Barrett's esophagus and high-grade dysplasia presented for EGD and biopsy in order to rule out invasive carcinoma. Endoscopic evaluation revealed a large hiatal hernia and an approximate 3 cm segment of Barrett's esophagus. There was a concerning focal area of ulceration which was removed via EMR.

**Figure 5:**
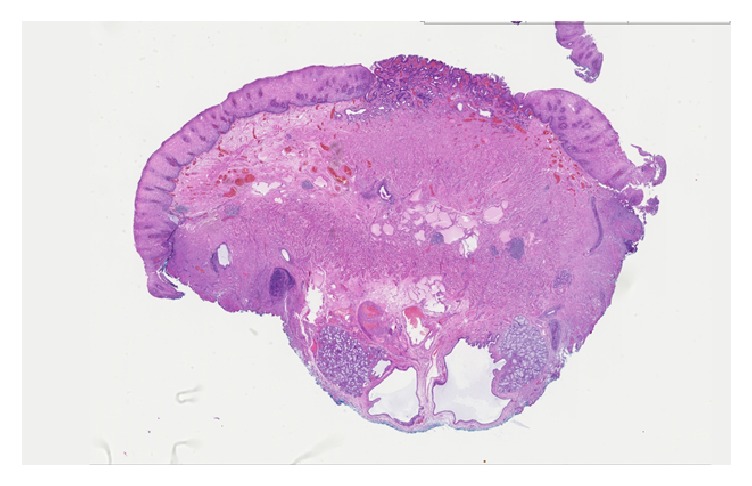
Low-magnification image showing focal high-grade dysplasia on the surface with ectatic venous channels and heterotopic minor salivary gland tissue in the deeper aspect.

**Figure 6:**
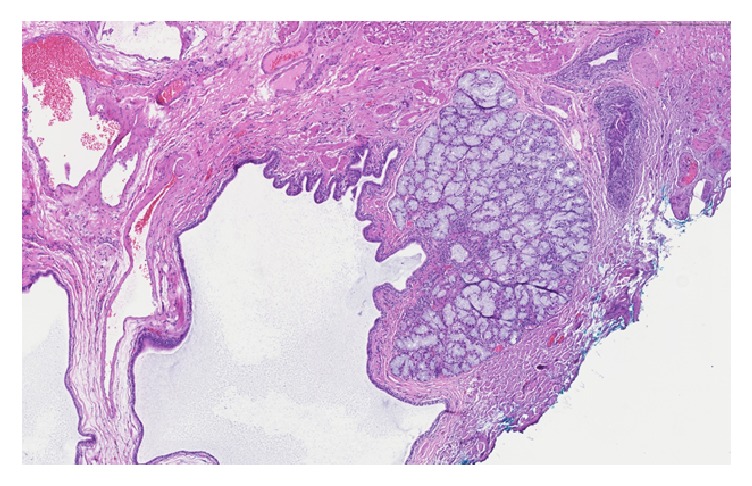
Higher magnification image showing the ectatic venous channels as well as a lobule of heterotopic minor salivary gland.

Grossly, the specimen was one piece of tan-white soft tissue, measuring 0.7 x 0.4 x 0.2 cm. Microscopic evaluation revealed focal high-grade dysplasia adjacent to squamous epithelium in the middle portion of the specimen. In the deeper aspect of the tissue, there were cavernous and ectatic venous channels as well as a few prominent lobules of minor salivary glands with cystification in the ducts (Figures 5 and 6). The patient was treated with radiofrequency ablation.

## 3. Discussion

Salivary gland heterotopia in the GI tract is a rare entity. The heterotopic tissue can form a mass, choristoma, which can present as a mass on imaging or as a hypoechoic mucosal or submucosal lesion on EUS, mimicking the presentation of carcinoma. Other possible differential diagnosis includes gastrointestinal stromal tumor (GIST), schwannoma, leiomyoma, ectopic pancreas, cyst, lipoma, and carcinoid. This review will focus on the clinical features, endoscopic/sigmoidoscopic findings, histopathology, ancillary studies, pathogenesis, treatment, and prognosis of salivary gland heterotopias/choristoma based on the 6 cases published in the literature (Table 2) with the addition of the 3 cases reported here for a total of 9 cases.

Salivary gland choristoma in gastrointestinal tract occurs mainly in adults but has been identified in an age range from 5 to 61 years. It occurs more often in males than it does in females, with 7 cases (78%) occurring in males. The race of the patients was not reported in most cases. It can occur in any location in the GI tract with reports of occurrence in the rectal area, rectal diverticulum, jejunum, esophagus, and, now, gastroesophageal junction. The clinical presentation depends on the location. Rectal cases can present as a rectal protrusion/mass, rectal bleeding, anal pain, and sphincter spasm. Cases in the GE junction either present with chronic reflux or are asymptomatic. One case presented in the esophagus with symptoms of belching, regurgitation, and abdominal pain. The youngest reported case was a 5-year-old girl who presented with severe, intermittent abdominal pain and postprandial vomiting due to a salivary choristoma in the jejunum.

The gross appearance is usually that of a single, tan-pink mucosal to subserosal nodule or pedunculated mass, ranging from 1 to 2 cm in size. Microscopically, mixed serous and mucinous glands with salivary ducts resembling submaxillary or sublingual salivary glands are seen.

All the reported cases were treated with either sigmoidoscopic or endoscopic resection, except for the case of the 5-year-old Nigerian girl who underwent exploratory laparotomy and wedge resection due to the unavailability of endoscopic procedures. There were no reports of complications or recurrences. However, the follow-up intervals varied. There was only one report of malignant transformation in the GI. However, this phenomenon has been described in multiple cases of salivary gland heterotopias in the head and neck.

Salivary gland choristoma in the GI tract can form a mass, which can present as a mass on imaging or as a hypoechoic mucosal or submucosal lesion on EUS, mimicking the presentation of a carcinoma. The authors do not suggest that it should be on top of the differential list, however, clinicians should be aware of its occurrence to prevent delay in diagnosis and inappropriate procedures and treatments for the patient.

## Figures and Tables

**Table 1:** Location, clinical features, endoscopic findings, and histopathology of salivary gland heterotopias at the gastroesophageal junction.

	**Age/Sex**	**Location**	**Clinical**	**Endoscopy**	**Histopathology**
Case 1	44m	GE Junction	8 month history of gastro-esophageal reflux disease (GERD).	Polypoid shaped, hypoechoic mass measuring 1 cm in size and confined to the deep mucosa and submucosa	Prominent mucus glands with chronic inflammation consistent with heterotopic salivary glands surrounded by prominent adipose tissue and congested vessels in the submucosa (Figures 1 and 2).

Case 2	62 m	GE Junction	History of hypothyroidism, Sjogren's syndrome, Raynaud syndrome, chronic GERD and grade 3 esophagitis	Dilated esophagus with an irregular z-line and 2 small islands of salmon colored mucosa immediately proximal to the GE junction	Focal mild acute erosive esophagitis and basal hyperplasia that was consistent with reflux and detached fragment of salivary gland type glandular tissue with chronic inflammation consistent with heterotopic salivary gland tissue (Figures 3 and 4)

Case 3	72 m	GE Junction	Past medical history of Barrett's esophagus and high-grade dysplasia presented to rule out invasive carcinoma	Concerning focal area of ulceration which was removed via EMR	Focal high-grade dysplasia, cavernous and ectatic venous channels as well as a few prominent lobules of minor salivary glands with cystification (Figures 5 and 6)

**Table 2:** Location, clinical features, endoscopic/sigmoidoscopic findings, histopathology, and treatment of 6 cases of gastrointestinal salivary gland choristomas reported in the literature.

Source	Age/ Sex	Location	Clinical	Endoscopy/ Sigmoidoscopy	Histopathology/ IHC	Treatment
Shindo et al, 1972 [6].	24 m	Hemorrhoid	Severe bleeding by rectum of sudden onset, protrusion, and anal pain, large external and internal hemorrhoids and considerable spasm of the sphincter.	N/A	Ectopic gastric mucosa is of the acid-secreting type normally found lining the corpus and fundus of the stomach. This type of mucosa covers dilated hemorrhoidal veins in this specimen. In another area, proximal to the gastric mucosa, there is a microfocus of mixed serous and mucous glands such as are found normally in the submaxillary salivary glands or in the mucosal glands of the tracheobronchial tree.	Resection

Weitzner et al, 1983 [7].	61 m	Hemorrhoid	1x1 cm anal verge polyp	N/A	Lobules of serous and mucous glands and ducts typical of submaxillary glands with and adjacent hyperplastic polyp	Simoidoscopic removal

Downs-Kelly et al, 2003 [8].	31 m	Rectal diverticulum	Intermittent rectal bleeding, small mass on the rectal wall. An ultrasound of the rectum revealed a 2 cm mass.	Sigmoidoscopy revealed a single diverticulum and an extramucosal mass in the lower rectal segment	In the submucosal region, multiple foci of serous and mucinous glands and ducts resembling salivary gland tissue were present. Some of these foci were associated with a chronic inflammatory cell infiltrate consisting primarily of lymphocytes.	The patient underwent local rectal excision of the 2x 2x 0.8 cm polypoid mass.

Maffini et al, 2012 [9].	55f	Large bowel	Colorectal carcinoma screening	A pedunculated polypoid lesion of 1 cm situated at 19 cm from the anal verge, resembling a submucosal lipoma without other mucosal alterations	A small aggregate of acinar glands with mixed mucous–serous features in the submucosa and an intercalated duct composed of a double layer of cells – epithelial and myoepithelial – that reached the mucosal surface. The glands were positive for lysozyme antibody and negative for pancreatic amylase, S-100 protein, chromogranin, and synaptophysin	Endoscopic resection

Olajide, 2013 [10].	5f	Jejunum	Severe, intermittent abdominal pain with occasional postprandial vomiting	A pale yellow subserosal lesion was seen in the antimesenteric border of the jejunum about 45 cm from the duodeno-jejunal junction.	Submucosal tissue with lobules of serous glands with central lumen, reminiscent of salivary glands. The glands are composed of benign epithelial cells with regular nuclei and ample eosinophilic cytoplasm.	Wedge resection

Wang et al, 2014 [11].	60 m	Esophagus	Belch, regurgitation, and abdominal pain	1.2 × 1.0 cm mucosal protuberant lesion situated 38 cm from the incisors	Salivary gland tumor, partly basal cell adenoma, partly with the structure of adenoid cystic carcinoma, the glands were positive for CD117, P63, PDGFR, P53, Ki-67, CEA, P-CK, Vimentin, PAS, S-100, Calponin, and CK5/6.	Endoscopic resection
